# Digital technologies to support adolescents with depression and anxiety: review

**DOI:** 10.1192/bja.2022.3

**Published:** 2023-07

**Authors:** Rhys Bevan Jones, Faris Hussain, Sharifah Shameem Agha, Bryony Weavers, Mathijs Lucassen, Sally Merry, Paul Stallard, Sharon Anne Simpson, Frances Rice

**Affiliations:** Senior Clinical Research Fellow in the Division of Psychological Medicine and Clinical Neurosciences, Cardiff University, and a psychiatrist with Cwm Taf Morgannwg University Health Board, Wales, UK.; Academic Foundation Year 2 doctor with Cwm Taf Morgannwg University Health Board, Wales, UK.; Lecturer with Cwm Taf Morgannwg University Health Board and an honorary lecturer in the Division of Psychological Medicine and Clinical Neurosciences, Cardiff University, Wales, UK.; Research assistant in the Division of Psychological Medicine and Clinical Neurosciences, Cardiff University, Wales, UK.; Senior lecturer in mental health in the Department of Health and Social Care, The Open University, UK, and an honorary senior lecturer in psychological medicine at the University of Auckland, New Zealand.; Professor in Child and Adolescent Mental Health in the Faculty of Medical and Health Sciences at the University of Auckland, New Zealand.; Professor of Child and Family Mental Health in the Department for Health at the University of Bath, England, UK.; Professor of Behavioural Sciences and Health in the Medical Research Council/Chief Scientist Office (MRC/CSO) Social and Public Health Sciences Unit, University of Glasgow, Scotland, UK.; Professor of Developmental Psychopathology in the Division of Psychological Medicine and Clinical Neurosciences, Cardiff University, Wales, UK.

**Keywords:** Digital, depression, anxiety, adolescence, support

## Abstract

Depression and anxiety are common in adolescents, but most affected will not get any formal help. Digital mental health technologies (i.e. resources and interventions to support and improve mental health) are a potential way to extend the reach and increase adolescents’ access to therapies, at a relatively low cost. Many young people can access the internet and mobile technologies, including in low- and middle-income countries. There has been increased interest in integrating technologies in a range of settings, especially because of the effect of the COVID-19 pandemic on adolescent mental health, at a time when services are under pressure. This clinical review gives an overview of digital technologies to support the prevention and management of depression and anxiety in adolescence. The technologies are presented in relation to their technological approaches, underlying psychological or other theories, setting, development, evaluations to date and how they might be accessed. There is also a discussion of the potential benefits, challenges and future developments in this field.

## LEARNING OBJECTIVES

After reading this article you will be able to:

- identify digital programmes developed and evaluated to prevent and manage depression and anxiety in adolescents
- understand the underlying psychological theories and technological approaches utilised, and how technologies might be developed, evaluated and accessed in health, education and other settings
- appreciate the potential benefits, challenges and future developments in this field.

Depression and anxiety are common in adolescents, but most individuals requiring support do not receive any formal help (Potter 2012; Neufeld 2017). The reasons for the high levels of unmet clinical need in adolescent depression and anxiety are complex. However, one way of improving access to evidence-based resources and interventions is via digital mental health technologies. Indeed, digital mental health has been identified as a key area of future clinical practice, in particular for preventing and treating adolescent depression and anxiety (Brent 2015). Digital mental health technologies include resources and interventions that are delivered via devices such as computers, tablets or smartphones to support and improve mental health. Digital technologies have been highlighted as particularly valuable given their potential to improve reach and to ease access to psychotherapy, at a relatively low cost (Stasiak 2016).

Digital technologies can vary in design (e.g. website-based or delivered via apps) and can include a range of digital elements (e.g. games, online activities and chatbots) to help personalise and better meet the range of needs and preferences of users. Some of these technologies are supported by contact with a healthcare provider or other professional. At one end of the human support continuum, this can include a service that is delivered by a clinician digitally in ‘real time’ (e.g. therapy provided via videoconferencing). At the other end of the continuum, resources and interventions are ‘pure self-help’ and therefore involve no human support to an individual user.

Most young people in high-income countries have access to the internet and mobile technologies. For instance, in the UK only 12% (*n* = 700 000) of those aged between 11 and 18 years reported having no access to the internet at home from a computer or tablet (Office for National Statistics 2019), and by the end of 2020 around 94% of homes had internet access (Ofcom 2021). Many young people in low- and middle-income countries (LMICs) can also access the internet, where there might be a lack of alternative face-to-face approaches (Naslund 2017). More recently, there has been increased interest in implementing remote assessments as well as technologies in a range of settings – especially because of the effects of the COVID-19 pandemic on adolescent mental health, at a time when services are under particular pressure (Ford 2021; Newlove-Delgado 2021).

There is growing evidence to support the use of some technologies in preventing and treating adolescent anxiety and depression (Reyes-Portillo 2014; Stasiak 2016; Hollis 2017; Grist 2019), and the National Institute for Health and Care Excellent (NICE) guidelines for the identification and management of depression in children and young people recommend digital cognitive–behavioural therapy (CBT) in the management of mild depression (NICE 2019).

This review article is written with both practitioners and researchers in mind, especially those interested in child and adolescent mental health and novel resources and interventions. The overarching aim is to provide a clinical overview of digital mental health technologies to support the prevention and treatment of depression and anxiety in adolescents.

## Review method

Relevant articles were identified through online searches of two databases (Medline and PsycInfo via Ovid) with no restriction regarding publication dates up until March 2021. The key search terms used were: ‘teen*’ or ‘young’ or ‘youth’ or ‘adolescen*’ or ‘child*’ or ‘paediatric’ or ‘pediatric’ AND ‘internet’ or ‘social media’ or ‘telemedicine’ or ‘cellular phone’ or ‘mobile phone’ or ‘smartphone app*’ or ‘mobile app*’ or ‘digital’ or ‘web-based’ or ‘website*’ or ‘ehealth’ or ‘e-health’ or ‘mhealth’ or ‘m-health’ or ‘online’ or ‘computer*’ AND ‘depress*’ or ‘mood’ or ‘anxiety’. We also searched reviews, guidelines and reference lists.

Studies were included if they:

- were articles with information on the evaluation of digital technologies to prevent and manage depression and anxiety (diagnosis or symptoms) in adolescents (primarily aged 13 to 18 years old); and
- were papers published in English in a peer-reviewed journal.

Papers were excluded if the technologies were developed for adults or primarily for physical health and well-being, or were diagnostic, screening, monitoring, communication or data management digital tools.

The titles and abstracts were screened (via Mendeley) by F.H., S.S.A., B.W. and R.B.J. (*n* = 5503 papers). Then the full texts of the studies on the included digital technologies (*n* = 33 technologies) were read in more detail and the relevant information was summarised. Key papers on the evaluation of each technology were cited. Key authors were contacted for further information, especially on how to access each technology. As this was an educational and descriptive review, and not a formal systematic literature review, an inclusive and pragmatic approach was taken in summarising the relevant papers. We have supplemented the key findings from the literature with our own reflections as practitioners or researchers working in the field.

## General characteristics of the identified digital technologies

The 33 identified digital technologies are presented in Table 1. Most of them target depression symptoms alone or together with anxiety symptoms, but a few are for anxiety only. Many are designed to be used in different contexts, including clinical and community settings, whereas others are designed to be integrated into specific settings, such as schools. The majority of the technologies are based, at least in part, on the principles of CBT, and many are informed by a combination of psychological or other theories, including positive psychology and interpersonal psychotherapy (IPT). NICE (2019) guidelines cite three digital CBT programmes for mild depression – specifically SPARX (Merry 2012), Stressbusters (Wright 2020) and Grasp the Opportunity (Ip 2016) – and these three are described in later sections.

**TABLE 1:** Digital technologies to support adolescents with depression and anxiety

Digital programme (reference, country)	Mental health difficulties and setting (e.g. clinic, school)	Psychological and other theories	Technological approaches and delivery	Stakeholder involvement in programme development	Evaluation and findings	Access (based on information from authors, papers and online sources)
**Structured and modular approaches**						
MoodGYM (Calear, 2009; Australia	Depression, anxiety. Any setting	CBT	Interactive modules, workbook. Stand-alone	Interviews/focus groups: young people	Cluster RCT: 1477 school students. Reduction in levels of anxiety at 6 months (*v*. waiting list), effects on depressive symptoms less strong	Available for free in Australia, subscription fee elsewhere: moodgym.com.au
CATCH-IT (Gladstone 2018; USA)	Depression. Community, clinics	CBT, IPT	Interactive modules. Supported	Groups/questionnaires: young people, practitioners	RCT: 369 young people in primary care. Reduction in depression symptoms in both arms, but no difference (*v*. general health education, attention control), may prevent depressive episodes in those with subsyndromal depression	Information: catchit-public.bsd.uchicago.edu
Grasp the Opportunity (Ip 2016; Hong Kong)	Depression. Schools	CBT	Interactive modules. Stand-alone	Questionnaires/focus groups/discussions: young people, parents, teachers, practitioners	RCT: 257 students. Reduction in depressive symptoms at 12 months (*v*. anti-smoking website, attention control)	Not available outside of research projects
CURB (Bansa 2018; USA)	Depression. Primary care	CBT, IPT	Interactive modules. Supported	Surveys/workshops: young people, parents, practitioners	Feasibility study: challenges and successes in screening and implementing in urban ethnic minority primary care settings	Information: checkbot-201623.appspot.com/index
Stressbusters (Smith 2015; Wright 2020; England)	Depression. Schools, community, clinics	CBT	Interactive sessions, videos. Stand-alone	Surveys/focus groups: young people, practitioners, designers	Smith 2015: RCT of 112 young people in schools. Improvement in depression and anxiety symptoms at 6 months (*v*. waiting list) Wright 2020: RCT of 139 young people from community and clinics. Improvement in both arms, but no difference at 12 months (*v*. self-help websites, attention control). Earlier study showed reduced depressive symptoms at 4 months	Not available outside of research projects
Think, Feel, Do (Stallard 2011; England)	Depression, anxiety. Clinics, schools	CBT	Interactive sessions, videos. Supported	Focus groups: children and young people, designers	Pilot RCT: 20 young people, improvement on 7 subscales compared with improvement on 3 subscales in waiting-list control. Moderate to high satisfaction	Not available outside of research projects
PST (Hoek 2012; The Netherlands)	Depression, anxiety. Schools, community	Problem-solving therapy	Lessons, exercises, email support. Supported	Adapted from PST programme for adults by authors	RCT: 45 young people. Overall improvement in depression and anxiety for both arms, but no difference (*v*. waiting list)	Not available outside of research projects
LEAP Project (Rickhi 2015; Canada)	Depression, well-being. Community, schools, clinics	Psychotherapeutic spiritual principles (e.g. gratitude, compassion)	Interactive modules, videos, relaxation techniques, journal. Stand-alone	Assignments/meetings/workshops/focus groups: young people, parents, practitioners, educators	Pilot RCT: 31 young people. Reduced severity of depression (*v*. waiting list)	Not available outside of research projects. Reformulated as BreathingRoom: breathingroom.me
iCBT (Topooco 2019; Sweden)	Depression. Community	CBT	iCBT modules, assignments, reflection. Supported	Surveys, text/oral feedback: users, practitioners	RCT: 70 young people. Improvements in depression symptoms post-treatment (*v*. minimal attention control), sustained at 12 months	Not available outside of research projects
iPDT (Lindqvist 2020; Sweden)	Depression. Schools, clinics, community	Psychodynamic psychotherapy	Modules, exercises, therapist feedback. Supported	Adapted from psychodynamic programme for adults by authors	RCT: 76 young people. Reduced depression and anxiety symptoms at 6 months (*v*. online supportive contact)	Not available outside of research projects
DAD Programme (Moeini 2019; Iran)	Depression. Schools	Social cognitive theory	Modules, videos, animations, slides. Supported	Interviews/focus group: young people	RCT: 128 female students. Improved depression symptoms at 12 weeks (*v*. control group).	Not available outside of research projects
BRAVE-ONLINE (Spence 2011; Australia)	Anxiety. Any setting	CBT	Interactive sessions, animations, games. Stand-alone and supported versions	Surveys/focus groups: CYP, practitioners	RCT: 115 young people. Similar improvements in anxiety diagnoses and symptoms compared with face-to-face therapy at 12 months (and greater than waiting list)	BRAVE Self-Help available free in Australia: brave4you.psy.uq.edu.au BRAVE Therapist-Assisted not available outside research projects. Information: brave-online.com
Cool Teens/ ChilledOut Online (Wuthrich 2012; Stjerneklar 2019; Australia)	Anxiety. Schools, community, clinics	CBT	Multimedia information, activities, homework. Supported	Questionnaires: young people	Cool Teens: RCT: 43 young people. Reduced number and severity of anxiety disorders post-treatment (*v*. waiting list), sustained at 3 months. ChilledOut: RCT: 70 young people. Outperformed waiting-list condition on diagnostic severity and anxiety symptoms post-treatment (sustained at 12 months)	Not available outside of research projects or centres with service agreements. Information: chilledout.org.au
Camp Cope-A-Lot (Pryor 2021; USA)	Anxiety. Any setting	CBT	Interactive sessions, animation, cartoon characters, homework. Supported	Adapted from Coping Cat programme by authors	Controlled trial: 27 CYP with anxiety and ASD. Reduced anxiety compared with digital social skills programme	Information: copingcatparents.com/Camp_Cope_A_Lot
**Gamification, serious games and avatars**						
SPARX (Merry 2012; New Zealand)	Depression. Any setting (especially primary care)	CBT	Gamification, avatars. Stand-alone	Workshops/focus groups: young people, families, clinicians, designers, cultural advisors	RCT: 187 young people in primary healthcare. Not inferior to TAU (primarily face-to-face counselling) on depressive symptoms at 3 months	Available online and via app stores for free in New Zealand: sparx.org.nz
The Journey (Stasiak 2014; New Zealand)	Depression. Schools	CBT	Gamification, videos/animation. Stand-alone	Workshops: young people, designers	Pilot RCT: 34 students. Feasible, acceptable and reduction in depressive symptoms (*v*. computerised psychoeducation)	Not available
SPARX-R (Perry 2017; Australia)	Depression. Schools	CBT	Gamification. Stand-alone	Interviews/focus groups: young people, families, practitioners	RCT: 540 students. Reduced depression symptoms at 6 months(*v*. online attention control)	Not available outside of research projects. Information: sparx.org.nz
Rainbow SPARX (Lucassen 2015; New Zealand)	Depression. Schools, primary care	CBT	Gamification, avatars. Stand-alone	Questionnaires/focus groups: young people	Open pilot trial: 21 sexual minority youth. Acceptable, feasible, and reduction in depressive symptoms	Not available outside of research projects. Information: sparx.org.nz
Pesky/Mindful gNATs (Tunney 2017; van der Meulen 2019; Ireland)	Anxiety, depression. Primary care, clinics	CBT, mindfulness	Gamification. Supported	Focus groups: CYP, practitioners, designers	Tunney 2017: two-armed qualitative study: 93 CYP. Six themes identified, technology can offer ‘rich experience’. van der Meulen 2019: naturalistic deployment study: 21 therapists, 95 young people. Three themes identified, supports real-world delivery	Available for licence fee (some resources free): peskygnats.com and mindfulgnats.com
**Moderated group, social media and messaging**						
Master Your Mood Online (van der Zanden 2012; The Netherlands)	Depression, anxiety. Community	CBT	Mood measure, facilitated sessions, chat function. Supported	Adapted from face-to-face intervention and Coping with Depression course by authors	RCT: 244 young people from community. Reduced depression and anxiety symptoms at 3 months (*v*. waiting list), sustained at 6 months.	Information: gripopjedip.nl
MEMO (Whittaker 2017; New Zealand)	Depression. Schools	CBT	Text messages, videos/ animations. Stand-alone	Focus groups: young people, practitioners, mHealth experts	RCT: 855 students. More from MEMO CBT group felt it helped them to be more positive and to get rid of negative thoughts (*v*. MEMO control). No effect on depressive symptoms	Not available
Rebound/ MOST (Rice 2018; Australia)	Depression. Clinics	CBT, mindfulness, positive psychology, social support	Social media-enabled platform. Supported	Workshops/focus groups/ consultations: young people, families, professionals, writers/artists, designers	Pilot study: 42 young people. Acceptable, feasible and safe	Not available outside of research projects. Information: orygen.org.au/Clinical-Care/Clinical-services/Moderated-Online-Social-Therapy
SOVA (Radovic 2018; USA)	Depression, anxiety. Clinics, community	Social support, psycho-education	Moderated social media. Supported	Interviews/focus groups: young people, parents, advocates, professionals	Feasibility study: 96 young people, established feasibility and usability	Available for free at: sova.pitt.edu wisesova.pitt.edu
**School-based technologies**						
Climate Schools (Teesson 2020; Australia)	Depression, anxiety, substance misuse. Schools	Psycho-education, CBT, harm minimisation	Interactive modules, illustrated storylines. Stand-alone (with teacher component)	Focus groups: young people, practitioners, designers	Cluster RCT: 6386 students. Combined intervention led to increased knowledge and decreased anxiety symptoms at 30 months (*v*. health education, active control)	Available to schools for free in Australia: rebranded as OurFutures ourfutures.education Adapted materials available in UK (climateschools.co.uk) and USA (climateschools.com)
Smooth Sailing (O'Dea 2021; Australia)	Depression, anxiety. Schools	Step-wise approach: psycho-education, CBT, school counselling	Stepped-care, interactive modules. Supported	Surveys/interviews/focus groups: young people, counsellors, GPs, parents	Cluster RCT: 1841 students. Marginal difference in help-seeking intentions at 12 weeks (*v*. school-as-usual control).	Not widely available outside research projects. Information: blackdoginstitute.org.au/research-projects/smooth-sailing
e-couch Anxiety and Worry programme (Calear 2016; Australia)	Anxiety. Schools	Psycho-education, CBT	Interactive modules. Stand-alone	Developed from existing programmes by researchers and practitioners, input/feedback from consumers	Cluster RCT: 1767 students. Externally supported *v*. teacher-supported *v*. waiting list. No differences on mental health or well-being	Available for free in Australia; with subscription fee elsewhere: ecouch.com.au
**Emerging interactive and personalised technologies**						
Shamiri-Digital (Osborn 2020; Kenya)	Depression, anxiety. Schools	Growth mindset, gratitude, value affirmation	Interactive exercises (single session). Stand-alone	Feedback from recent high school graduates, authors	RCT: 103 students. Reduced depressive symptoms at 2 weeks (*v*. study-skills control)	Available for free: outside research projects. thrive-online.shamiri.institute
Depis.Net (Anttila 2021, Finland)	Depression. Clinics, schools	Self-determination theory	Sessions, exercises, mood/sleep diary, support system. Supported	Interviews: young people, practitioners	Quasi-experimental study: 151 students. No difference in health outcomes (*v*. control group). Encouraging results on adherence and acceptance	Not available outside research projects
Quest-Te Whitianga (Christie 2019; Fleming 2019; Bevan Jones 2020a; New Zealand)	Anxiety, depression. Any setting	CBT, positive psychology, mindfulness, interpersonal skills	Modular activities, gamification. Stand-alone	Interviews/focus groups/ workshops: young people, designers, practitioners	Acceptability testing: support from young people, Māori and Pasifika people and clinicians	Not available outside of research projects
HABITs (Thabrew 2018; Merry 2020; New Zealand)	Emotional health, substance use. Schools, community	CBT, positive psychology, harm minimisation	Different user groups: games, chatbots, intrinsic motivators; digital ecosystem. Stand-alone	Surveys/focus groups: young people, practitioners, cultural advisors	Acceptability/usability testing: young people with similar mental health needs and backgrounds had diverse preferences, including gamified and serious/simple interface	Not available outside of research projects
MoodHwb (Bevan Jones 2020b; Wales)	Depression. Clinics, schools, community	Psycho-education, CBT, social support	Illustrations/animations, profile builder, mood diary, goal-setting. Stand-alone	Interviews/workshops/focus groups: young people, parents/carers, practitioners, designers	Feasibility evaluation; 44 young people, 31 parents/carers. Acceptable and feasible	Not available outside of research projects
Smartteen (Srivastava 2020; India)	Depression. Clinics	CBT	Interactive presentation, quizzes, vignettes, diary. Supported	Focus groups: young people, practitioners	Pilot RCT: 21 young people. Feasible, acceptable and reduced depressive symptoms	Not available outside research projects
Anxiety Coach (Whiteside 2019; USA)	Anxiety. Clinics	Exposure therapy	Interactive activities, parent-coached. Supported	Developed by authors	Feasibility study: 4 therapists, 8 CYP. Feasible and acceptable	Information: anxietycoach.mayoclinic.org/anxiety

ASD, autism spectrum disorder; CBT, cognitive–behavioural therapy; CYP, children and young people; GP, general practitioner; iCBT, internet-based cognitive–behavioural therapy; iPDT, internet-based psychodynamic psychotherapy; IPT, interpersonal psychotherapy; RCT, randomised controlled trial; TAU, treatment as usual; *v*., versus (compared with).

A range of digital elements are used in technologies that aim to help ensure the programme is relevant, personalised, flexible and engaging for the user. These include interactive exercises, gamification, avatars, video clips, animations, social media components, user messaging and chatbots. Many of the programmes include several of these elements, and they are categorised here primarily in terms of the digital approach taken. Some are developed to be used independently as ‘stand-alone’ technologies (unguided), whereas others are supported by practitioners (guided). Most technologies had elements of co-design with potential users in their development, particularly those produced in recent years (Bevan Jones 2020a).

In summarising the technologies, we have prioritised the programmes that have been evaluated via randomised controlled trials (RCTs), and we include other programmes with emerging evidence, in particular where there has been feasibility testing. Most technologies identified in our search are not yet available outside of research projects. However, we also refer to available technologies that did not meet the inclusion criteria. We discuss the overall effectiveness and challenges (including accessibility) of technologies later. Further information on each technology (e.g. screenshots) can be found on the websites and papers cited in Table 1 and by contacting the authors and developers. All programme characteristics (e.g. health difficulties targeted, settings, therapeutic and technological approaches, effectiveness, availability) can be considered when selecting which technologies meet the needs and preferences of young people in specific contexts.

## Technological approaches and underlying psychological theories

### Structured and modular approaches

#### MoodGYM

Early digital mental health technologies for adolescents have tended to be structured in modules or sections, and were often adapted from conventional face-to-face or established manualised approaches. One of the earliest online programmes for depression and anxiety to be trialled in young people is MoodGYM. This is a fully automated, interactive and self-directed modular CBT programme, accompanied by a workbook. It aims to change dysfunctional thoughts and beliefs, improve self-esteem and interpersonal relationships, and teach skills such as problem-solving and relaxation. MoodGYM was developed originally in Australia for young people and adults, but has been used and evaluated in several countries (e.g. Calear 2009) and translated into several languages. However, at present it is only available in English and German.

#### CATCH-IT, Grasp the Opportunity and CURB

Another early example of an internet-based programme for adolescents is CATCH-IT (Competent Adulthood Transition with Cognitive-behavioral, Humanistic and Interpersonal Training), which is a depression prevention intervention developed in the USA. This consists of interactive modules based on CBT, behavioural activation and IPT, and a component to provide support to parents. It is based on previously manualised and face-to-face approaches (Gladstone 2018).

CATCH-IT has also been adapted for different settings and countries. For example, Grasp the Opportunity is a translation and adaptation developed for Hong Kong Chinese adolescents and (as previously highlighted) is cited in the NICE (2019) guidelines for depression in children and young people. Grasp the Opportunity has modules with stories to illustrate relevant concepts, and it aims to improve negative cognition, reduce negative behaviours, strengthen resilience and reinforce positive behaviours (Ip 2016). CURB (Chicago Urban Resiliency Building) is a cultural adaptation of CATCH-IT for socio-economically disadvantaged African American and Latino adolescents (Bansa 2018).

#### Stressbusters, Think, Feel, Do and PST

Stressbusters is a modular CBT programme for depression developed from a manualised treatment programme in the UK, and it is also cited in the NICE (2019) guidelines for depression in children and young people. Sessions include video clips, animations, graphics and printed resources. Users progress through the programme in a linear manner, and the intervention also includes homework tasks with an associated mood diary. It has been evaluated in schools, communities and mental health services (Smith 2015; Wright 2020).

Another early CBT programme developed in the UK is Think, Feel, Do (Stallard 2011). This is a guided programme for emotional problems and consists of six sessions. The sessions focus on emotion recognition and management; linking thoughts, feelings and behaviour; identifying and challenging negative thoughts; and problem-solving. Think, Feel, Do is interactive and involves quizzes, video clips, music and animation. PST (problem-solving therapy) is another early structured technology for both depression and anxiety, and was evaluated in The Netherlands (Hoek 2012).

#### LEAP Project, iCBT, iPDT and DAD

More recent examples of modular interventions for depression include the LEAP Project, informed by spiritual principles (e.g. forgiveness, gratitude, compassion) from Canada (Rickhi 2015), and iCBT and iPDT (psychodynamic psychotherapy) technologies – both evaluated in Sweden (Topooco 2019; Lindqvist 2020). The DAD programme is a depression course informed by social cognitive theory, which has been evaluated with female school students in Iran (Moeini 2019).

#### BRAVE-ONLINE, Cool Teens, ChilledOut Online and Camp Cope-a-Lot

BRAVE-ONLINE is one of the first online interventions specifically for anxiety and has been trialled with both children and young people. This intervention is based on CBT and has interactive sessions, animations and games. It was originally developed in Australia, but has been evaluated in several countries (Spence 2011). Cool Teens is another example of an early CBT-based technology for anxiety in Australia (Wuthrich 2012), and has been adapted into ChilledOut Online (Stjerneklar 2019). Camp Cope-a-Lot is an early CBT programme (adapted from Coping Cat) evaluated with both children and young people, including adolescents with anxiety and autism spectrum disorder, in the USA (Pryor 2021).

### Serious games, gamification and avatars

**‘**Serious games’ are a form of ‘applied games’ and are in essence computerised games designed for ‘serious’ purposes, for example to improve mental health outcomes. ‘Gamification’ in digital technologies refers to gaming elements used to enhance an intervention's appeal, outside of a fully bespoke ‘serious game’. Both serious games and gamification have been highlighted as ways to potentially increase the impact of mental health interventions (Fleming 2017).

#### SPARX, The Journey, SPARX-R and Rainbow SPARX

**FIG 1:**
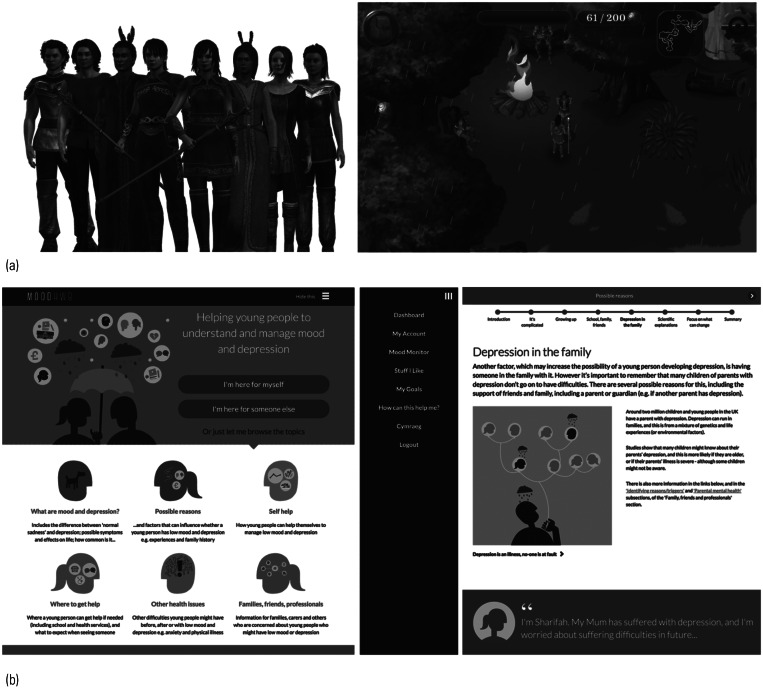
Examples of screenshots and characters from digital technologies. (a) SPARX (Merry 2012). (b) MoodHwb (Bevan Jones 2020b).

SPARX (Smart, Positive, Active, Realistic, X-factor thoughts) is CBT in serious game format developed in New Zealand (Merry 2012) (Fig. 1(a)). The user chooses an avatar and travels through seven provinces in the SPARX fantasy world dominated by GNATs (Gloomy Negative Automatic Thoughts). The user undertakes a series of challenges to restore balance and interacts with a ‘guide’, who puts the game into context. SPARX was informed by an earlier CBT intervention, The Journey (Stasiak 2014), which was a prototype serious game intervention, also developed in New Zealand. SPARX has been evaluated in primary care (Merry 2012), and subsequently refined and tested with school students in Australia using SPARX-R, the resiliency version (Perry 2017), and refined for sexual minority youth using Rainbow SPARX (Lucassen 2015).

#### Pesky gNATs and Mindful gNATs

Pesky gNATs (a wordplay on ‘negative automatic thoughts’) is a computer game to support CBT for anxiety and low mood. This was originally designed for children and can be played during face-to-face therapy sessions with a practitioner. Users navigate a three-dimensional world and meet characters who introduce CBT concepts using audio, animation and video, and can learn behavioural activation, relaxation and mindfulness skills. There is online training for therapists, and evaluations include a naturalistic deployment study (van der Meulen 2019). Mindful gNATs is a related gamified programme with more focus on mindfulness training (Tunney 2017).

### Moderated group, social media and messaging platforms

Moderated group, social media and messaging platforms have been used in various contexts to support young people's mental health.

#### Master Your Mood Online

Master Your Mood Online is a group CBT course for depression and anxiety in adolescents (and young adults), derived from the Coping with Depression course. Cognitive restructuring of thinking patterns is at the core of the six-session course. It is facilitated by a practitioner and has been evaluated in mental healthcare agencies in The Netherlands (van der Zanden 2012).

#### MEMO

MEMO is a multimedia universal depression prevention intervention delivered by mobile phone. Key messages derived from CBT are delivered via text messages, video diaries and messages, and ‘mobisodes’ of a cartoon. Delivery approaches were influenced by social cognitive theory and marketing principles. The content was designed to appeal to Māori and Pasifika adolescents, and it has been evaluated in schools in New Zealand (Whittaker 2017).

#### Rebound and SOVA

Rebound is a depression programme within MOST (Moderated Online Social Therapy), a social media-enabled mental health platform developed in Australia. It is based on CBT, mindfulness and positive psychology, and promotes social support. Rebound integrates peer-to-peer online social networking, individually tailored interactive psychosocial interventions/modules, and expert mental health and peer moderation, and has been evaluated in a pilot study (Rice 2018).

SOVA (Supporting Our Valued Adolescents) is a moderated social media website for young people with depression and anxiety, developed in the USA. It includes a blog with positive content (e.g. quotes, videos), psychoeducation, social media guidance and links to existing resources, and there has been feasibility testing (Radovic 2018).

### School-based technologies

#### Climate Schools Combined, Smooth Sailing and the e-couch Anxiety and Worry programme

Some programmes have been developed to be integrated in schools or educational settings. For example, Climate Schools Combined is a universal intervention developed to prevent substance use and mental health problems, particularly depression and anxiety, in young people. The programme is based on psychoeducational, CBT and harm minimisation approaches, and consists of interactive modules and illustrated ‘comic-book’ storylines. It has been evaluated in schools in Australia (Teesson 2020).

Smooth Sailing is a universal school-based mental health service for depression and anxiety in secondary school students. This uses a stepped-care approach informed by mental health help-seeking theories. A website assists in screening young people and allocates them to face-to-face school counselling or online multimedia psychoeducational and CBT approaches, at various intensities and follow-ups. This has been evaluated in Australian schools (O'Dea 2021).

The e-couch Anxiety and Worry programme is another intervention based on psychoeducation and CBT. It comprises six sessions, including three ‘toolkits’. There is a ‘CBT toolkit’ on addressing cognitive aspects of worrying, a ‘relaxation toolkit’, which includes mindfulness meditation exercises, and a ‘physical exercise toolkit’. It has also been evaluated in schools in Australia (Calear 2016).

### Emerging interactive and personalised technologies

Several technologies for depression and anxiety in adolescents are in development or undergoing evaluation, taking advantage of emerging digital elements (e.g. chatbots) to increase interactivity and personalisation, so that the technologies are engaging and meet the needs and preferences of users.

#### Shamiri-Digital and Depis.Net

Shamiri-Digital is an innovative single-session intervention for depression, anxiety and well-being. This consists of modules on growth, mindset, gratitude and value affirmation, particularly in response to various challenges. This was adapted originally from a group-based form, and the digital format has been evaluated in Kenya (Osborn 2020). Depis.Net is a ‘web-based support system’ for depression based on self-determination theory. It has sessions on well-being, home and family, rights and responsibilities, and depression and its treatment. It is monitored by a practitioner, and has been evaluated in clinics and schools in Finland (Anttila 2021).

#### Quest-Te Whitianga and HABITs

The SPARX team in New Zealand has also developed the Quest-Te Whitianga app for anxiety and depression. This incorporates CBT, positive psychology, mindfulness and promotes interpersonal skills. Its development was informed by that of SPARX, and it takes a modular approach with a selection of activities and games on different ‘islands’. For example, there is a gratitude journal on the ReMind Island and a relaxation and mindfulness activity on the ReLax Island (Christie 2019).

The Quest-Te Whitianga app will be integrated into a wider platform called HABITs (Health Advances through Behavioural Intervention Technologies) (Thabrew 2018; Merry 2020). This is a ‘digital ecosystem’ to support young people with their emotional health and substance use. It is designed to host and evaluate a range of digital health interventions based on CBT, positive psychology and a harm minimisation approach, including serious games and links to other sources of help.

#### MoodHwb, Smartteen and Anxiety Coach

MoodHwb is a programme for young people with depression and their families/carers, developed in Wales (Fig. 1(b)). It is delivered in Welsh and English and has illustrations, animations, and interactive and personalised components. It is based on psychoeducation, CBT, behavioural activation, positive psychology and interpersonal theory. It aims to promote self-help, help-seeking where appropriate and social support (Bevan Jones 2020b).

Other emerging technologies include Smartteen, a CBT-informed programme for depression evaluated in India (Srivastava 2020), and Anxiety Coach, which is based on exposure therapy and developed in the USA (Whiteside 2019).

### Other relevant technologies

#### Starship Rescue and Bounce Back

Other technologies might also be helpful in preventing or managing depression and anxiety in adolescents, although it is beyond the scope of this review to cover these in detail. Some have been developed for specific forms of anxiety, such as social anxiety (Tillfors 2011), or anxiety associated with physical conditions (e.g. Starship Rescue; Thabrew 2018). Other technologies focus on specific contexts, for example Bounce Back is a behavioural activation programme to help support individuals in disaster settings, who have experienced depression and other difficulties (Davidson 2014).

#### BlueIce, Sleep Ninja and Sleepio

Specialist technologies targeted at other primary mental health difficulties might also be helpful for depression or anxiety. For example, BlueIce which is a dialectical behaviour therapy (DBT) and CBT-informed technology for self-harm, developed in the UK (Stallard 2018). Examples of CBT-informed technologies for insomnia include Sleep Ninja, which has a ‘ninja’ chatbot trainer, and has been developed in Australia (Werner-Seidler 2019), and Sleepio, which is presented by a cartoon therapist, and has been developed in the UK (Cliffe 2020).

#### BiteBack, Reach Out Central, and Professor Gooley and the Flame of Mind

Technologies developed for general youth mental health often include information on depression and anxiety. For example, BiteBack is a youth positive psychology programme (Manicavasagar 2014), Reach Out Central is a CBT-based ‘serious game’ for young people (Webb 2008) (both developed in Australia), and Professor Gooley and the Flame of Mind is a game-based programme developed in Hong Kong (Huen 2016).

#### The Aroha chatbot and KeepCool

Technologies have also been developed and adapted to support young people during the COVID-19 pandemic and beyond – including the supportive Aroha chatbot on HABITs in New Zealand (Merry 2020) and KeepCool in the UK (kcl.ac.uk/research/keepcool).

### Finding available technologies

Available technologies (some of which might not have been identified in our search) are also listed on websites, such as PsyberGuide (onemindpsyberguide.org) and Beacon (beacon.anu.edu.au). These have different approaches to reviewing and recommending technologies. The NHS closed its Apps Library site in late 2021 and will link to recommended apps throughout the NHS website (nhs.uk/mental–health/children). There are also mental health websites with information on youth depression and anxiety, for example YoungMinds (youngminds.org.uk), the Royal College of Psychiatrists (rcpsych.ac.uk/mental-health/parents-and-young-people), the National Centre for Mental Health (ncmh.info/resources/) and the Lowdown (thelowdown.co.nz).

## Overall benefits and effectiveness of digital technologies

Systematic reviews of digital technologies for adolescents (e.g. Hollis 2017) have shown that there is more support for technologies for depression and anxiety than for other problems, such as neurodevelopmental difficulties. Reviews suggest that digital CBT interventions are particularly promising (Grist 2019) and, as noted earlier, NICE guidelines have cited three digital CBT technologies (SPARX, Stressbusters, Grasp the Opportunity) for mild depression (NICE 2019).

Of the 33 technologies identified in our review (summarised in Table 1), 18 were evaluated via effectiveness RCTs. Nine of these were developed for depression, six for depression and anxiety, and three for anxiety alone. Most of these studies showed an improvement in mental health outcomes (e.g. symptoms, disorders) at least in the short term, in those using the technologies (compared with controls), or the technologies performed at least as well as face-to-face approaches. The studies showed that digital technologies can also help with other mental health-related outcomes, including knowledge, stigma and help-seeking behaviour.

Other potential benefits of digital technologies include the increase in reach and access to therapies at relatively low cost, flexibility of use and increased personal choice (Table 2). It is also possible to tailor programmes to address diversity in the user group. Furthermore, digital programmes can help young people to communicate and connect with practitioners and each other, and to reduce feelings of isolation, using devices and media that they use in their daily lives.

**TABLE 2:** Potential benefits and challenges of digital mental health technologies for adolescents

Potential benefits	Potential challenges	Suggestions to overcome challenges
Increased reach Ease of access via internet and mobile technologies Range of digital formats and design elements Flexibility of use and personal choice, greater convenience Variety of psychological theories utilised e.g. CBT, behavioural activation, IPT Varied usability, e.g. school, clinical and community settings Can be used to address a range of presentations and outcomes, e.g. symptoms of depression and anxiety	Methodological limitations of studies Limited user engagement and adherence Poor treatment completion rates Concerns over data protection and privacy Lack of programme availability, few effective programmes available outside of clinical trials Different legal requirements regarding approvals and data storage across international boundaries Difficulties addressing the needs of diverse user groups (e.g. working as well for specific cultures and other underserved subgroups) Meeting high expectations of young people related to modern technologies, e.g. that serious games are sufficiently contemporary Difficulties adequately identifying potential harm or adverse effects while using technologies (e.g. recognising mental health difficulties and ensuring suitable follow-up is provided) Negative experiences might affect future help-seeking behaviour Resources and training required when user support is needed	Co-design and co-development with potential users Rigorous and appropriate evaluation Awareness and information about programmes, their target audience and their effectiveness Moderation and ongoing oversight of digital interventions and platforms Therapist support, where appropriate, by practitioners with a range of clinical experience Use of security measures, e.g. passwords, usernames and data protection policies Improved efficiency of research cycles for designing and more rapidly evaluating programmes Prescribing digital interventions relative to certain characteristics, e.g. age, gender and clinical presentation Adapt digital interventions for specific settings, subgroups and contexts

CBT, cognitive–behavioural therapy; IPT, interpersonal psychotherapy.

## Potential challenges of digital technologies

Although the studies are promising, there are several key challenges in this field, including methodological limitations (Hollis 2017; Grist 2019). There is a need for more technologies that have been co-developed and evaluated rigorously according to research frameworks (e.g. Craig 2008). Many of the studies available are small and primarily undertaken by the programme developers. Follow-ups are lacking and therefore little is known about the longer-term benefits of digital interventions. Even technologies recommended by NICE have limited evidence, for example the most recent evaluation of Stressbusters did not find a positive effect at 12 months (Wright 2020).

Other challenges include low user engagement, uptake and adherence to these programmes. Uptake and adherence might be improved by providing support by practitioners, which might require further time, resources and training. However, a systematic review found that support can be provided by practitioners with various levels of clinical training (Baumeister 2014).

There are concerns related to data protection, security, and privacy or confidentiality – which can affect the user's engagement and trust in the technology (Hollis 2017; Fleming 2018). This is especially relevant if a technology enables communication between users, such as with social media or forums, and moderation is important in this context. Other elements to ensure compliance with data protection regulations include the use of usernames and passwords to log in, locks, data encryption and secure servers. There is a risk that the use of technologies might cause harm, especially where there is an absence of rigorous evaluation and data on effectiveness or safety by independent research groups.

Most of the studies and technologies identified in this review were delivered only in English and were created in high-income countries. However, there is an increasing interest in the use of digital technologies in LMICs, where many have access to the internet and mobile devices, but lack formal face-to-face approaches to mental healthcare (Naslund 2017). There is also a lack of studies involving those with neurodevelopmental difficulties, intellectual disabilities and other specific difficulties.

Another significant challenge is that many of the interventions for young people cited in Table 1 are not yet widely available or have not been evaluated outside of a specific country and setting – as interventions developed in one setting might not be transferrable to another. There might also be different legal requirements regarding government approvals and data storage requirements across international boundaries. There is a divide between programmes that are evaluated but not available and those that are readily available but not evaluated independently (Wasil 2019). This may be partly because of the resources required to complete studies and the long time frames associated with research cycles, for instance from securing funding for the initial research to intervention development and evaluation, then to the implementation of the technology, if this occurs (Craig 2008). This contrasts with the fast pace of progress in technology generally.

Therefore, in practice, young people and families/carers seeking online support for depression or anxiety might access general mental health websites that provide written information (e.g. on NHS webpages) or programmes developed primarily for adults (e.g. Headspace, Calm), and some of these involve subscription costs.

## Addressing user needs and preferences

There are several other factors to consider when choosing the most appropriate digital programme for depression or anxiety, as noted earlier. Young people and families/carers need to consider whether the technology is appropriate given the user's characteristics, such as their age, development, gender identity, and the nature and severity of their difficulties. In a scoping study, Fleming et al (2019) concluded that younger adolescents who experienced stress or low mood were more likely to be interested in interactive and gamified digital interventions that were specifically targeted to support them, whereas older adolescents who experienced these difficulties were more interested in ‘direct-to-the-point’, serious approaches with a clean and clear design.

Technologies have also been developed or adapted to engage with specific cultures and subgroups (e.g. Ip 2016; Bansa 2018). Considerations in this context include language, text, iconography, symbols, metaphors, colours, characters and, in some cases, the general principles or philosophy of the technology. For example, SPARX was created by a game company led by a Māori director and included co-design work facilitated by a Māori clinician among *rangatahi* (adolescents). This work was overseen by a Kaumātua (respected Māori elder) as well as a cultural advisory group. These efforts were employed to create a programme that would be maximally appealing and relevant to Māori (as well as non-Māori) adolescents (Shepherd 2015). SPARX has also been translated into several languages, including Dutch, French, Japanese and Inuktitut (Nunavut, Canada), and adapted for sexual minority youth, as noted earlier (Lucassen 2015).

Another consideration is whether the technology might be more relevant for specific settings, such as clinics, communities and schools. In addition, young people will have their individual preferences regarding the content and design of technologies and their overall approach (e.g. social media, chatbot). Some might prefer to engage only with face-to-face therapy or self-help in printed literature format. Therefore, it is important that young people have a choice, where possible, regarding how they access support.

## Future developments

Given the fast pace of digital technology and culture, there are a number of possible future developments in digital mental health practice and research. Technologies are likely to become more complex, flexible and personalised, including the use of avatar-based virtual therapy. For instance, a study is underway that explores whether a virtual reality intervention provides a more engaging user-directed experience than a comparable purely web-based intervention (Schleider 2019). Although avatars might facilitate communication with verbally uncommunicative adolescents, professional attitudes and concerns about a lack of information technology (IT) expertise, time or effectiveness need to be addressed if these are to be widely used (Falconer 2019).

Collaboration and co-design with young people and other stakeholders are recommended throughout the research cycle of technologies, including the development, evaluation and implementation stages (see stakeholder involvement in Table 1). This is to ensure that technologies meet the users’ needs and preferences, and that they are potentially more engaging, feasible and effective. This might also lead to increased uptake and adherence. There are additional challenges here, and further guidance and research is required in this field (Bevan Jones 2020a).

To help with the faster translation of findings into the community, more flexible and rapid models of development and evaluation in the ‘real world’ will be needed, for example using digital ecosystems that support rapid retesting as the technologies evolve (Rice 2018; Merry 2020). However, there should still be a rigorous approach to development and evaluation, with no harm to participants and users and an assessment of cost-effectiveness where possible. More research is required, in particular into the implementation phase of technologies (Craig 2008), specifically how to get programmes from the ‘lab’ into different settings. To help to achieve these aims, there could also be greater collaboration and sharing of knowledge between those developing and evaluating technologies (including research and commercial groups) across the world.

Although there are studies involving certain subgroups, young people with intellectual disabilities and specific difficulties will have particular needs and preferences, and should be involved further in research. Digital technologies might be particularly helpful for those who have difficulties with verbal communication. More technologies could be developed for children and younger adolescents, and those who might provide support, such as peers, families, carers and practitioners. Further attention is also required on developing or adapting technologies to tackle the mental health difficulties arising from the COVID-19 pandemic/crisis across the world, including in LMICs (Ford 2021; Newlove-Delgado 2021).

## Conclusions

A number of digital technologies have been developed and evaluated to support young people with depression and anxiety, and some have been cited in guidelines for the management of depression in young people (NICE 2019). There are potential benefits of using technologies, although there are also challenges to overcome – for example related to usage and adherence, evaluation, availability, and security and confidentiality. Further research and guidance are required in this field regarding all phases of the research cycle. This is to ensure that technologies are made in collaboration with potential users and evaluated using appropriate and possibly novel methods – so that they are accessible, acceptable, feasible and effective in supporting young people and their families, carers, friends and practitioners.

## Data availability

Data availability is not applicable to this article as no new data were created or analysed in this study.

## MCQs

Select the single best option for each question stem

1. The National Institute for Health and Care Excellence (NICE) guidelines recommend the following for management of depression in children and young people:
  - digital interpersonal therapy
  - digital art psychotherapy
  - digital family therapy
  - digital cognitive–behavioural therapy
  - digital psychodynamic therapy.
2. The three digital technologies cited by the NICE guidelines for depression in children and young people include:
  - MEMO
  - MoodGYM
  - Pesky gNATs
  - Stressbusters
  - Think, Feel, Do.
3. The NICE guidelines for depression in children and young people recommend digital cognitive–behavioural therapy for 12- to 18-year-olds with:
  - mild depression
  - moderate depression
  - severe depression
  - psychotic depression
  - any severity of depression.
4. Which of the following is one of the earliest digital technologies developed and evaluated to support depression and anxiety in young people?
  - HABITs
  - MoodGYM
  - Smooth Sailing
  - Quest-Te Whitianga
  - MoodHwb.
5. Which of the following is an example of a ‘serious game’?
  - SPARX
  - Stressbusters
  - Rebound
  - Think, Feel, Do
  - Master Your Mood.

MCQ answers 1 d  2 d  3 a  4 b  5 a
